# Against quantiles: categorization of continuous variables in epidemiologic research, and its discontents

**DOI:** 10.1186/1471-2288-12-21

**Published:** 2012-02-29

**Authors:** Caroline Bennette, Andrew Vickers

**Affiliations:** 1 Pharmaceutical Outcomes Research and Policy Program, University of Washington, Box 357630, Seattle, WA 98195-7630, USA; 2 Department of Epidemiology and Biostatistics, Memorial Sloan-Kettering Cancer Center, 307 E 63 rd St, New York, NY 10065, USA

## Abstract

**Background:**

Quantiles are a staple of epidemiologic research: in contemporary epidemiologic practice, continuous variables are typically categorized into tertiles, quartiles and quintiles as a means to illustrate the relationship between a continuous exposure and a binary outcome.

**Discussion:**

In this paper we argue that this approach is highly problematic and present several potential alternatives. We also discuss the perceived drawbacks of these newer statistical methods and the possible reasons for their slow adoption by epidemiologists.

**Summary:**

The use of quantiles is often inadequate for epidemiologic research with continuous variables.

## Background

Epidemiology is often introduced using examples in which both exposure and outcome are considered in binary terms: research participants are defined as having, say, lung cancer or not, and being smokers or not, and then the proportion of smokers compared between cases and controls. Many exposures, however, are inherently continuous. Indeed, in the classic case-control study on smoking and lung cancer[1], Doll and Bradford-Hill report results both for cases and controls in terms of proportion of smokers and by "amount of tobacco consumed", grouping into several different categories such as 1 - 4, 15-24 or 50 + cigarettes per day. In contemporary epidemiologic practice, it is more customary to group continuous variables into quantiles - most often tertiles, quartiles or quintiles - based on the exposure's distribution. In one recent study, for example, researchers examining the link between dietary fat and breast cancer grouped fat intake into quintiles. They reported that women in the highest quintile of fat intake were 11% more likely to get breast cancer than women in the lowest quintile[2]. As another example, surgeon annual caseload was found to be significantly associated with the survival of patients after an acute myocardial infarction[3]. The authors reported that the 30-day mortality rate was 13.5% for physicians in the lowest quartile of volume (5 or fewer cases per year) compared to 11.8% for physicians in the highest quartile (more than 24 cases annually).

A number of researchers have commented on the disadvantages of categorization in epidemiologic studies[4]. Many associations can be tested using linear models and practicable alternative methods for handling non-linear relationships have been broadly developed and validated in recent years. Yet despite these methodological advancements and calls for the abandonment of percentile-based categorization [4,5], the epidemiologic community continues to rely heavily on the use of quantiles as a primary means of analyzing and presenting results. For example, in a recent issue of *The American Journal of Epidemiology *(October 2009, volume 170, number 8), four of six papers with a continuous exposure used some form of percentile-based categorization; only two kept the variable as continuous.

Quantiles appear intuitively appealing to epidemiologists as they can be thought of in terms of low, medium and high risk groups. Moreover, the association between exposure and outcome can be described in terms of a relative risk between these groups. However, these perceived benefits are outweighed by several important problems that arise when a continuous variable is categorized, particularly if data dependent quantiles are used to form categories. Here we summarize the previous research on the topic and address possible concerns about the use of alternative statistical approaches.

## Discussion

### Analysis

Categorization of continuously distributed exposure variables is associated with three problems: first, it involves multiple hypothesis testing with pairwise comparisons of quantiles; second, it requires an unrealistic step-function of risk that assumes homogeneity of risk within groups, leading to both a loss of power and inaccurate estimation; and third, it leads to difficulty comparing results across studies due to the data-driven cut points used to define categories.

### Multiple testing

Investigators often use the lowest quartile or quintile as the reference category and test whether subsequently higher categories are associated with increased risk of an outcome. For example, a recent study examined whether height was associated with risk of Alzheimer's disease [6]. To test this hypothesis, the authors grouped height into quartiles and separately tested whether the proportion of subjects with Alzheimer's disease was significantly different between the lowest and each of the three highest quartiles; a p value was obtained for each of the three comparisons. As is well known, the chance of a false positive result is increased by multiple comparisons. In the study above, one of three comparisons among men was found to be statistically significant - that between men in the highest and lowest quartile of height - and was subsequently reported in the abstract; the two non-significant results were not mentioned. In another example, researchers reported a positive association between triglycerides and risk of endometrial cancer despite only showing that those in the highest quartile had higher risk compared to those in the lowest; no significant differences were seen for the other quartiles. Moreover, an association was found for only one of the four exposures analyzed, all of which were grouped into quartiles [7]. Naturally, this problem can be circumvented by conducting an overall test of significance. Yet the use of multiple groups in quantile-based analysis surely exacerbates the tendency to multiple testing, and this is clearly what is seen in the literature [6,7].

### Homogeneity of risk within categories

The assumption that risk is homogeneous within categories is often inappropriate, especially if the exposure distribution is skewed. Take, for example, a recent study reported that arsenic levels were not associated with risk of bladder cancer [8]. Using levels derived from toenail clippings, the investigators categorized men into quartiles based on the distribution of arsenic in the controls. Arsenic levels in the first three quartiles ranged from 0.014 to 0.161 μg/g; levels in the highest quartile ranged from 0.161 to 17.5 μg/g. By grouping arsenic exposure in this manner, the authors implicitly assume that 0.17 and 17 μg/g of arsenic have identical effects on bladder cancer risk, a 100-fold difference in exposure. The assumption that risk does not vary within categories naturally has implications for statistical power. Ignoring intracategory variation and ordering means throwing away information, and is prone to reduce a study's power to detect an association, particularly when, as is common, the exposure distribution is asymmetric[5].

**Figure 1:**
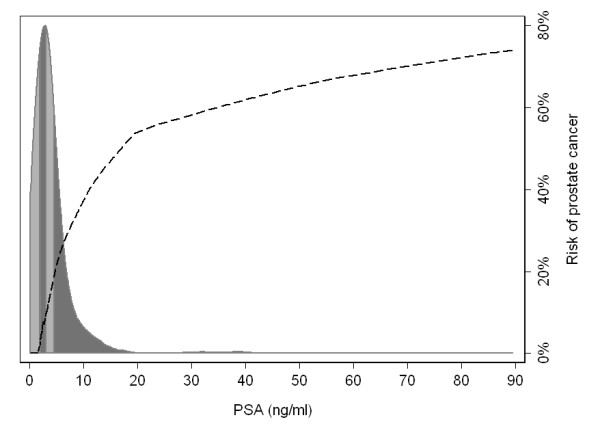
**The risk of prostate cancer by level of PSA, with the distribution of PSA levels (ng/ml) among a population-based sample**. The shading in the distribution gives the quartiles of PSA.

The assumption of homogeneity with risk groups also leads to inaccurate estimation. Figure 1 illustrates a typical risk of prostate cancer by PSA, a biomarker, in a sample of older men. Similar to the arsenic data, the range of PSA within the highest quartile, illustrated by the darker grey shading, is much larger than the range between the lowest three quartiles. It is implausible to assume a constant risk within the highest quartile - between ~5 ng/ml and 100 ng/ml - and it is clear that reporting only the mean risk by quartile would lead to grossly inaccurate estimates of risk. In this example, the mean risk in the top quartile of PSA was 30%. For men between the 75th and 85th centile - 10% of the population - true mean risk is only 22%, considerably less than that estimated by the quantile approach; for patients in the top 5%, true risk is 54%, nearly double the risk of the highest quartile as a whole.

### Comparison between studies

Where categorization is based on quantiles, it becomes difficult to compare results across different studies. For example, two recent papers described the effect of hospital volume on mortality rates after a surgical treatment for colon cancer. One study, using data from the Surveillance, Epidemiology and End Results-Medicare linked database, found a significant association between hospital volume and 30-day survival[9]; the other, using data from hospitals in Ontario, failed to find this association[10]. It is difficult to reconcile these inconsistent findings because the measure of exposure - quartile of hospital volume - was dependent on the data set used for analysis. In the first study, the lowest quartile of hospital volume was defined as 57 or fewer cases over a 6- year period whereas for the second study, the lowest quartile included hospitals performing fewer than 61 cases in a 3- year period, more than a twofold difference in volume. This would be analogous to comparing two studies on the association between poverty and health, when poverty was defined as an annual income of $22,000 in one and $47,000 in the other.

### Alternatives to categorization

The natural approach to determining an association between a continuously distributed exposure and a binary outcome is simply to analyze the exposure variable in its raw, continuous state using a linear regression model. Basic regression approaches assume a linear association between exposure and the log odds of outcome, but there exist straightforward methods to model non-linear relationships. In the case of a single explanatory variable and outcome, locally-weighted regression ("lowess") is a robust modeling method; where adjustment for covariates is required, several alternative curve-fitting strategies exist. Among them, splines and fractional polynomials are easily implemented in multivariable regression[11,12].

**Figure 2:**
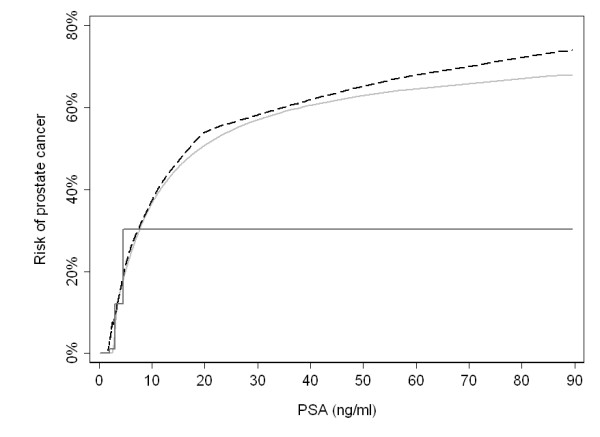
**Prostate cancer risk by PSA (black dashed line), with predicted risks using either cubic splines (light gray solid line) or quartiles (dark gray solid line)**.

As an example, Figure 2 illustrates the true risk of prostate cancer by PSA level, as well as the average risk within quartiles and a regression model using restricted cubic splines. The predicted risk from the spline regression model results in a much better approximation of the true risk function than the quartiles. In particular, the use of quartiles failed to detect the continued increase in risk for subjects at the highest PSA levels; by definition, quartiles cannot detect an increase in risk beyond the 75th centile of the exposure. The estimated risk derived from the quartiles differed from the true absolute risk by more than 5% for close to one third of subjects; in contrast, the predicted risk from the spline model differed by more than 5% for about one in 15, close an 80% relative reduction in the risk of an inaccurate estimate.

### Perceived problems with non-linear modeling

Both cubic splines and fractional polynomials are easy to implement with most statistical software - one simply adds splined terms or transformed variables into the regression model - and both provide several important benefits: a more realistic estimation of the exposure-risk relationship and the ability to test directly for non-linearity. However, despite these advantages, several perceived drawbacks appear to have limited their widespread adoption by epidemiologists.

The first argument against non-linear modeling of continuous variables is that doing so fails to provide a parsimonious description of the exposure-disease relationship, and this hinders communication of the results to the public and other scientists. Yet this argument holds both for non-linear modeling and the use of categorization: when quartiles or quintiles are used to describe a relationship between an exposure and an outcome, the relationship is described by three or four separate estimates. In the study of height and Alzheimer's disease, for example, the investigators categorized height into quartiles and therefore obtained three separate estimates of association[6]. The choice to report only one comparison is arguably an inappropriate way to simplify their findings; indeed, it ignores the data from 50% of the study participants.

**Figure 3:**
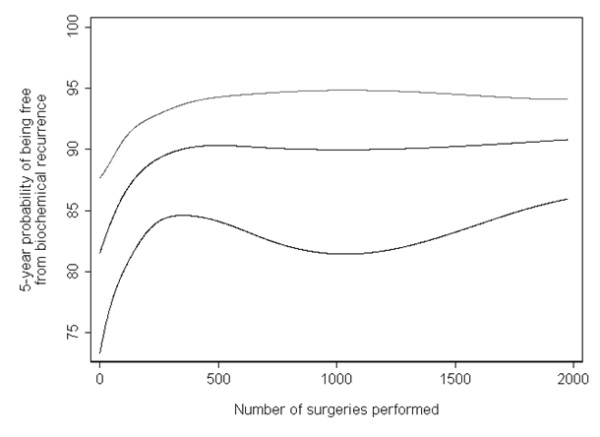
**The learning curve for cancer control after radical prostatectomy**.

Moreover, the main analyses from a regression model involving non-linear terms can be described concisely and clearly. For example, one of us has previously reported on the impact of the surgical experience on recurrence rates after surgery for prostate cancer [13]. As the association between surgeon experience and outcome was hypothesized to plateau after a certain level of practice, non-linear terms for experience were included in the regression model. Instead of reporting a series of hazard ratios comparing the risk of recurrence for patients treated by surgeons in the higher quintiles of experience compared those in the lowest, the results from the main analysis were presented graphically. Figure 3 shows the probability of recurrence by surgical experience and clearly illustrates the dramatic improvement and subsequent plateau of recurrence rates seen with increasing experience. Clinically meaningful comparisons derived from the non-linear model can concisely describe the association: we reported the difference in absolute risk of recurrence for a typical patient treated by a surgeon who had performed 10 procedures and for a surgeon who had performed 250 prior procedures. These values were chosen after consultation with surgeons and were intended to reflect meaningful levels of experience; the estimates are obtained from the model including non-linear terms, not from a categorization approach. In contrast, quantiles rely on data driven cut points. The study of arsenic exposure provides a good illustration: it seems abstruse to compare risk between 0.014 and 0.161 μg/g; there is no evidence that 0.161 μg/g is biologically relevant. In our view, analyses of continuous variables can be presented in readily meaningful terms; in fact, we would argue that clinically relevant comparisons are often more easily understood and useful than the estimates derived from data-driven quantiles.

Another argument against regression techniques involving non-linear terms is that the resulting models are prone to overfit [14]. While the increased flexibility of fractional polynomial or spline regression models may create spurious dips or inflection points, the random variability associated with the estimation of each quantile can similarly lead to spurious findings. A good example of this effect comes from a study of sex hormones and prostate cancer risk. Using the lowest quartile as the reference category, the odd ratios for successively higher estradiol quartiles were reported as 0.53 (95% Confidence Interval [CI]: 0.33, 0.85), 0.40 (95% CI: 0.23, 0.70) and 0.56 (95% CI: 0.32, 0.98). As a result, the authors concluded that the association between estradiol and prostate cancer risk was non-linear[15]. Yet while central estimates across quantiles do not follow a linear trend, the confidence intervals around each estimate are wide, and it is hard to tell exactly how much evidence of non-linearity is provided by the data. With formal non-linear modeling, conversely, evidence of non-linearity can be tested directly, by a joint test on the non-linear terms.

Approaches using splines or fractional polynomials to model non-linearity are also open to criticism for being overly sensitive to the placement of knots or choice of polynomial terms. Although it is no doubt true that, in some cases, the exposure-risk relationship is sensitive to decisions about modeling, we would argue that categorization is comparably sensitive to the choice of cut points. For example, a study by Filardo et al. found that the association between body mass index [BMI] and in-hospital mortality after coronary artery bypass graft surgery was strongly influenced by the way in which BMI was categorized[16]. A model that included cubic splines revealed a strong non-monotonic relationship in which patients with very low and very high BMI were at increased risk; on the other hand, findings were inconsistent when BMI was categorized, depending on whether tertiles, quartiles or quintiles were used. Clearly, use of non-linear modeling is not invulnerable to poor analytic practice, such as failure to consider overly influential outlying observations. As ever, good modeling depends on close collaboration with those who have good content knowledge -and who can therefore assess whether a curve or subset of observations makes sense - use of appropriate regression diagnostics, and sensitivity analysis, to determine how curves change with alternative model specifications or removal of subsets of observations.

A final argument against the non-linear modeling to predict risk concerns application to case- control studies. Where the risk is fixed by design - such as a mean 25% risk in a case-control study with 3:1 matching - applying a regression model to a data set will lead to a misestimation of risk. However, this can be easily remedied by recalibration. A simple approach is to add a constant (sometimes described as a Bayes factor) to the linear prediction[17]. Alternatively, imputation approaches can be used to estimate levels of the exposure in controls not subject to sampling[18].

## Summary

We are far from the first to argue against the categorization of continuous variables[4,5] or advocate for the use of non-linear modeling of continuous variables[11,12,19]. Yet categorization, particularly by use of quantiles, remains extremely common in the epidemiologic literature. We hypothesize that this is largely due to historical precedent. Non-linear models are computationally intensive, and would have been extremely difficult to implement in the early years of modern epidemiology. Doll and Bradford-Hill had little choice but to categorize the continuous variable of smoking; however, with modern statistical methods and computing, there is no need to follow blindly in their wake.

We are not advocating for the complete abandonment of categorization. In fact, we often use quantiles in the preliminary assessment of exposure-outcome relationships [13,20]. But we go on to model these relationships directly, using non-linear terms. Doing so allows us to avoid implausible assumptions that risk does not vary within categories, and report results based on our entire sample for clinically sensible cut-points that can be compared between different studies[13,20]. We feel that there are very few, if any, circumstances in which the use of quantiles would be the preferred method of reporting results. As such, we encourage other investigators to abandon use of categorization as a principal analysis in epidemiologic research.

## Abbreviations

CI: Confidence interval; BMI: Body mass index; PSA: Prostate-specific antigen.

## Competing interests

The author declare that they have no competing interests.

## Authors' contributions

AV conceived of the analysis. CS performed the analyses and drafted the manuscript. AV reviewed for important intellectual content. All authors have read and approved the final manuscript.

## Pre-publication history

The pre-publication history for this paper can be accessed here:

http://www.biomedcentral.com/1471-2288/12/21/prepub

## References

[B1] Doll R Hill AB Smoking and carcinoma of the lung; preliminary report Br Med J 1950 2 4682 739 748 10.1136/bmj.2.4682.739 14772469 PMC2038856

[B2] Thiebaut AC Kipnis V Chang SC Subar AF Thompson FE Rosenberg PS Hollenbeck AR Leitzmann M Schatzkin A Dietary fat and postmenopausal invasive breast cancer in the National Institutes of Health-AARP Diet and Health Study cohort J Natl Cancer Inst 2007 99 6 451 462 10.1093/jnci/djk094 17374835

[B3] Tu JV Austin PC Chan BT Relationship between annual volume of patients treated by admitting physician and mortality after acute myocardial infarction JAMA 2001 285 24 3116 3122 10.1001/jama.285.24.3116 11427140

[B4] Rothman K Greenland S Lash T Modern Epidemiology 2008 3 Lippincott Williams & Wilkins

[B5] Greenland S Avoiding power loss associated with categorization and ordinal scores in dose-response and trend analysis Epidemiology 1995 6 4 450 454 10.1097/00001648-199507000-00025 7548361

[B6] Petot GJ Vega U Traore F Fritsch T Debanne SM Friedland RP Lerner AJ Height and Alzheimer's disease: findings from a case-control study J Alzheimers Dis 2007 11 3 337 341 17851184 10.3233/jad-2007-11310

[B7] Lindemann K Vatten LJ Ellstrom-Engh M Eskild A Serum lipids and endometrial cancer risk: results from the HUNT-II study Int J Cancer 2009 124 12 2938 2941 10.1002/ijc.24285 19267407

[B8] Michaud DS Wright ME Cantor KP Taylor PR Virtamo J Albanes D Arsenic concentrations in prediagnostic toenails and the risk of bladder cancer in a cohort study of male smokers Am J Epidemiol 2004 160 9 853 859 10.1093/aje/kwh295 15496537

[B9] Schrag D Cramer LD Bach PB Cohen AM Warren JL Begg CB Influence of hospital procedure volume on outcomes following surgery for colon cancer JAMA 2000 284 23 3028 3035 10.1001/jama.284.23.3028 11122590

[B10] Simunovic M Rempel E Theriault ME Coates A Whelan T Holowaty E Langer B Levine M Influence of hospital characteristics on operative death and survival of patients after major cancer surgery in Ontario Can J Surg 2006 49 4 251 258 16948883 PMC3207572

[B11] Greenland S Dose-response and trend analysis in epidemiology: alternatives to categorical analysis Epidemiology 1995 6 4 356 365 10.1097/00001648-199507000-00005 7548341

[B12] Royston P A strategy for modelling the effect of a continuous covariate in medicine and epidemiology Stat Med 2000 19 14 1831 1847 10.1002/1097-0258(20000730)19:14<1831::AID-SIM502>3.0.CO;2-1 10867674

[B13] Vickers AJ Bianco FJ Serio AM Eastham JA Schrag D Klein EA Reuther AM Kattan MW Pontes JE Scardino PT The surgical learning curve for prostate cancer control after radical prostatectomy J Natl Cancer Inst 2007 99 15 1171 1177 10.1093/jnci/djm060 17652279

[B14] Weinberg CR How bad is categorization? Epidemiology 1995 6 4 345 347 10.1097/00001648-199507000-00002 7548338

[B15] Gann PH Hennekens CH Ma J Longcope C Stampfer MJ Prospective study of sex hormone levels and risk of prostate cancer J Natl Cancer Inst 1996 88 16 1118 1126 10.1093/jnci/88.16.1118 8757191

[B16] Filardo G Hamilton C Hamman B Ng HK Grayburn P Categorizing BMI may lead to biased results in studies investigating in-hospital mortality after isolated CABG J Clin Epidemiol 2007 60 11 1132 1139 10.1016/j.jclinepi.2007.01.008 17938055

[B17] Ulmert D Cronin AM Bjork T O'Brien MP Scardino PT Eastham JA Becker C Berglund G Vickers AJ Lilja H Prostate-specific antigen at or before age 50 as a predictor of advanced prostate cancer diagnosed up to 25 years later: a case-control study BMC Med 2008 15 6 6 18279502 10.1186/1741-7015-6-6 PMC2275744

[B18] Vickers AJ Gupta A Savage CJ Pettersson K Dhalin A Bjartell A Manjer J Scardino PT Ulmert D Lilja H A panel of kallikrein marker predicts prostate cancer in a large, population-based cohort followed for 15 years without screening Cancer Epidemiol Biomarkers Prev 2011 20 2 255 61 10.1158/1055-9965.EPI-10-1003 21148123 PMC3035761

[B19] Royston P Sauerbrei W Building multivariable regression models with continuous covariates in clinical epidemiology-with an emphasis on fractional polynomials Methods Inf Med 2005 44 4 561 571 16342923

[B20] Vickers AJ Savage CJ Hruza M Tuerk I Koenig P Martinez-Pineiro L Janetschek G Guillonneau B The surgical learning curve for laparoscopic radical prostatectomy: a retrospective cohort study Lancet Oncol 2009 10 5 475 480 10.1016/S1470-2045(09)70079-8 19342300 PMC2777762

